# Supplementary material for: Study on optimization measures for water level fluctuation of large water conveyance aqueducts
Source: PLoS One. 2025 Apr 1;20(4):e0320397. doi: 10.1371/journal.pone.0320397 (PMC11960963; doi:10.1371/journal.pone.0320397)
Supplement: Attachment — Submitted filename: Reply to authors.docx [file pone.0320397.s001.docx]

**Reply to the Authors**

**Title:** Optimization Measures for Mitigating Water Surface Fluctuations in Large-Scale Water Conveyance Aqueducts

**Manuscript No**: PONE-D-23-24085

**Authors:** Jian Chen; Yangyang Tian; Hongling Shi; Huijie Zhang

In this article, the authors reported a typical three-dimensional hydrodynamic model with optimization of the tail pier structure. The simulation results demonstrate that a conical tail pier can effectively eliminate water surface fluctuations, reduce turbulent energy dissipation rates at the exit transition section, and maintain a relatively stable Froude number and flow pattern. In addition, a 15 m long tapered tail pier incurred less head loss with a small tapered tail pier. The research reported in this article is technically relevant and has certain practical application value.

However, the paper is not well drafted. The authors are required to incorporate the following corrections/clarifications before the manuscript can be considered for publication.

1. **Abstract:** The abstract needs to do a better job of clarifying and justifying the significance of this study. What is the importance of doing this, and what is the novelty of this work?
2. **Introduction:** The authors do not provide a good bibliographical review of the current state of the art in this type of problem. We suggest that the authors conduct a good review and place the article in an international context. Please add some recent references in this context (the reference list only contain 20 articles). You need to better signify the importance of the problem and highlight the issues and their significance. The statement of aims is clear, but before this you need to add a gap analysis paragraph that justifies the need for your research.
3. **Analysis of simulation results:** Results and discussion suggestions are presented separately, and subheadings can be set for discussions under different working conditions.
4. **Figures:** The quality of all the figures needs improvement, and the target structure should be highlighted.
5. **References:** Please ensure that all references are relevant to the contents of the manuscript and that suitable recent references are added.
6. **Language:** Please review the English level and decide whether revisions are necessary.
