# Supplementary material for: Study on optimization measures for water level fluctuation of large water conveyance aqueducts
Source: PLoS One. 2025 Apr 1;20(4):e0320397. doi: 10.1371/journal.pone.0320397 (PMC11960963; doi:10.1371/journal.pone.0320397)
Supplement: Attachment — Submitted filename: Response to Reviewers.docx [file pone.0320397.s002.docx]

1. Abstract: The abstract needs to do a better job of clarifying and justifying the significance of this study. What is the importance of doing this, and what is the novelty of this work?

Modifications have been made accordingly.

2. Introduction: The authors do not provide a good bibliographical review of the current state of the art in this type of problem. We suggest that the authors conduct a good review and place the article in an international context. Please add some recent references in this context (the reference list only contain 20 articles). You need to better signify the importance of the problem and highlight the issues and their significance. The statement of aims is clear, but before this you need to add a gap analysis paragraph that justifies the need for your research.

Modifications have been made accordingly.

3. Analysis of simulation results: Results and discussion suggestions are presented separately, and subheadings can be set for discussions under different working conditions.

Modifications have been made accordingly.

4. Figures: The quality of all the figures needs improvement, and the target structure should be highlighted.

Modifications have been made accordingly.

5. References: Please ensure that all references are relevant to the contents of the manuscript and that suitable recent references are added.

Modifications have been made accordingly.

6. Language: Please review the English level and decide whether revisions are necessary.

Modifications have been made accordingly.

Reviewer #2: This work presents results from a study examining how tail pier structure could affect water level, energy loss, and water surface fluctuations in aqueducts. The authors test 4 different pier structures and compare the results to an assumed baseline pier shape to determine if there is a reduction in fluctuations. I recommend that the paper be rejected due to the limited scope, overstating of results, and lack of clarity in assumptions and methods. I have included some comments below that may help the authors in refining the content if they choose to modify and resubmit this paper.

Major comments

1. The authors do not clearly define several very important terms that appear repeatedly in the paper, e.g., Karman vortex street (KVS), tail pier, twisted surface. I recommend explaining these terms in the Introduction.It would also be beneficial to briefly describe the cross-section structure of the aqueduct shown in Figs. 2 and 5.

It has been introduced accordingly. A brief description has been provided.

2. The authors should present more instances of KVS occurring in aqueducts.

Relevant introductions have been added.

3. Lines 34-36: the design maximum flow is provided out of context. Has KVS occurred in this system? Has this system observed flows greater than 420 m3/s?

"KVS has occurred. The observed flow rate is not greater than 420."

4. Lines 75-77: Why is it important to look at a transition section with a twisted surface? Please provide more clear terminology. It would be useful to include an outline here of what will follow in the paper, i.e., the contents of each section and a brief description of your methodology, results, and metrics of comparison. As it stands, the flow of the paper is hard to follow.

A twisted surface can guide the fluid to pass through the transition section more smoothly, reducing fluid separation and vortex generation, thereby lowering energy loss.

By precisely designing the shape and angle of the twisted surface, the fluid flow path can be further optimized, enhancing the overall system efficiency.

5. In the Introduction, establish a clear gap in the literature that you are trying to address with this work. The authors begin to do this only in Section 3.2 (lines 205-207) but do not clearly explain what a "twisted surface" is or why it is important to model that.

"Relevant explanations have been added."

6. Figs. 2 and 5 are very unclear. Please improve the resolution of the image and choose a more clear font.

"The Figs has been replaced with clearer Figs."

7. In Section 2 you describe a model of an aqueduct while in Section 3 you mention that you validate the model with measurements taken from a "typical aqueduct". Is the model from Section 2 (as well as its parameters and boundary conditions) based on this aqueduct described in Model 3? If that is the case, please specify in Section 2 that you are building the aqueduct model to represent a very specific existing aqueduct. If the model is not based on a specific aqueduct, how did you obtain the boundary conditions?

The mathematical model is established based on the prototype of the Turuohe Aqueduct in the South-to-North Water Diversion Project.Relevant explanations have been added in Section 2.

8. This study only runs simulations for a very specific case (for which initial and boundary conditions are specified). Therefore, any conclusions from these results cannot be generalized or applied to other aqueduct models or flow regimes. Broad statements in the Conclusion describing "excellent performance" are without justification. Much more robust analyses for a wide range of initial conditions, flow conditions, and aqueduct structures should be tested. Additionally, please add text describing future extensions of this work/avenues of research in the Conclusion section.

This paper aims to explore the impact of different types of tail piers on eliminating the Karman vortex street phenomenon, with a focus on comparing the types of tail piers.

The conclusion section has added possibilities for future research.

9. What is the "strong water blocking effect?" (from line 311 and Fig. 14) and what causes it?

The "strong water blocking effect" (referenced from line 311 and Figure 14) refers to a phenomenon where the flow of water is impeded or restricted, resulting in a reduction in flow velocity, an increase in water level, or a change in flow direction. This effect is typically caused by physical obstacles, narrow passages, sudden changes in pipe diameter, or sharp variations in flow velocity.

In the context of Figure 14 and the related description, the strong water blocking effect may be due to changes in the geometry of the pipeline or container (such as sudden narrowing or widening), internal obstacles, or changes in the angle between the flow direction and the pipe wall. When water flow encounters these changes, it may be obstructed, leading to a decrease in velocity, an increase in water level, and potentially the generation of turbulence or vortices.

10. The PLOS data policy requires that authors must make their data fully available without restriction. Please consider hosting the model, real aqueduct topology and parameters, and code on a public repository such as Github or in the Supplemental Materials.

Minor comments:

1. Please standardize the spelling of Karman vortex street throughout the paper.

It has been processed in accordance with standards.

2. Fig. 11 is never described in the text.

The corresponding instructions have been added.

3. Figs. 8 and 9 are incorrectly numbered.

The change has been made.

4. Line 96: Was the 3D model really constructed at a scale of 1:1?

Yes, it is a mathematical model.

5. Fig 5: Fig 5 only shows the locations of Sections A and B, it does not show a clear layout of the measuring points of the model as suggested by the caption.

The locations of the measurement points are described in sufficient detail in the text.

6. Line 37: What does "slapping and pulling beams" mean?

The article has been revised to: "Slapping against support beams"."Slapping against support beams" refers to the water hitting and impacting the structural beams supporting the aqueduct.

7. Define CFD, RNG when they first appear in the text.

Relevant definitions have been added.

8. There are too many terms to describe the different tail pier structures - "model condition" (line 215), "working condition (Figs 6-9), "condition", "mode" (Section 4). Please standardize the terminology.

The terminology has been standardized.
