# Supplementary material for: Study on optimization measures for water level fluctuation of large water conveyance aqueducts
Source: PLoS One. 2025 Apr 1;20(4):e0320397. doi: 10.1371/journal.pone.0320397 (PMC11960963; doi:10.1371/journal.pone.0320397)
Supplement: Attachment — Submitted filename: Response_to_Reviewers_auresp_2.docx [file pone.0320397.s003.docx]

1.In the previous round of revisions, the authors were asked to standardize the spelling and capitalization of the critical "Karman Vortex Street" term throughout the paper, and while the authors claim to have addressed this issue in their responses, the same errors persist (line 15 in the abstract, line 40, line 95, line 98 ...). Please take care to standardize these spellings.

The corresponding content has been fully modified

2.Abstract line 22: "a conical tail pier performs better in eliminating water surface fluctuations": performs better than what other tail pier shapes?

The experimental results demonstrate that a conical tail pier performs better than prismy tail pier in eliminating water surface fluctuations.

3.Abstract line 26: "a shorter, 15-meter conical tail pier incurs less head loss": less head loss compared to what?

Furthermore, by comparing the total head loss at the outlet tapering section, it is found that a shorter, 15-meter conical tail pier incurs less head loss than 35-meter conical tail pier, indicating that a smaller conical tail pier is more effective.

4.The word "Conditional" has been spelled "Conditionl" throughout the paper

The corresponding content has been fully modified.

6.I suggest reformatting each reference very carefully. Perhaps consider using a reference management software to standardize the format. The formatting is very inconsistent and the references themselves are hard to track and sometimes wrong - for example, the first paper should be from "Chinese Journal of Nature" and not "Nature Journal."

I have re-formatted each reference, and now they all follow the same format. The references themselves can also be traced.

7. I think the introduction needs to be made much stronger and the authors should be very careful and clear with the details. For example, the "Tacoma Canyon Bridge" in reality is named the "Tacoma Narrows Bridge." Also, the current format of the introduction is difficult to follow - lines 56-62 are not connected to the KVS specifically.And in lines 71 and 79 - what is the "numerical simulation conditional" and the "physical conditional test"?You use the term "conditional" throughout the paper but I do not see a clear explanation of what that means.Finally, I think adding more studies, as the other reviewer suggested, is critical to improve the trustworthiness and quality of this paper.

"Tacoma Canyon Bridge" has been changed to "Tacoma Narrows Bridge."

The content here is intended to introduce the negative impact of the Karman vortex street phenomenon in the water conveyance process of a flume by using a specific engineering example, further illustrating the hazards of the Karman Vortex Streets.The subsequent content of the article also focuses on research based on this flume as the prototype.

These two respectively represent “numerical simulation model” and “physical model experiments”, and I have already made the corresponding modifications in the text.

To avoid misunderstanding, I changed "conditional" to "model"in the necessary places.

"I have added content regarding open-channel turbulence and water conveyance capacity, corresponding to lines 77 to 101 in the text."

The anisotropy of turbulence in open channels is caused by the combined effects of the solid boundary and the water surface. The near-wall region of the open channel is the turbulence production zone [2]. Near the wall, the turbulence intensity increases with the distance from the wall(y). Experiments show that in the viscous sublayer, the longitudinal turbulence intensity increases linearly with y [3], reaching its maximum value within the range of y = 10–20 [4,5]. In the absence of secondary flow, the turbulence intensity in all directions decreases with the distance from the wall in the outer region, which can be expressed by an exponential law [5]. When considering the effects of secondary flow, the free surface in the open channel suppresses the vertical fluctuating velocity, and this suppression effect decreases with the distance from the water surface. As a result, the vertical turbulence intensity near the water surface decreases, while the longitudinal and lateral turbulence intensities increase due to the redistribution of turbulence energy [6], which promotes the generation of secondary flow. It is important to note that the above discussion regarding the influence of the water surface on turbulence intensity is based on conditions where the Froude number is not large or the surface tension is sufficiently high to suppress water surface fluctuations. When surface fluctuations are present and the Froude number approaches 1, the turbulence intensity near the water surface increases[3]. Theoretically, it seems more reasonable to expect that the turbulence energy near the water surface should decrease, as large vortices near the surface collide with the water surface, causing deformation of the vortex core and suppressing the vertical turbulence scale [7]. The experimental results of Nakagawa et al.[4]also support this analysis. However, the experiments of Komori et al. [6] show a slight increase in turbulence energy near the water surface. Therefore, the variation of turbulence energy near the water surface remains inconclusive.

8.Line 56: "South to North Water Transfer Project" located where? Please remember that PLOS One has an international readership.

The relevant explanation has been added：

The Central Line Project of South-to-North Water Diversion draws water from the Danjiangkou Reservoir, passes through Henan and Hubei provinces, crosses the Yellow River, and delivers water northward to Beijing and other northern regions. It is a major water transfer project connecting southern and northern China.

9.Line 139: N-S is Navier Stokes equation?

Yes, I have made the modifications in the text.

10.In Table 1 and 2 - please add the unit of measure for measured and simulated water depth ([m] I presume)

I have added notes to the table.

12.Line 119: add a citation for Rhino software

Rhino is a powerful professional 3D modeling software developed by Robert McNeel & Assoc in the United States for PCs. It can be widely applied in fields such as 3D animation production, industrial manufacturing, scientific research, and mechanical design.

13. The paper contains two many modeling specifics (e.g., lines 107 - 115, line 175- 178, lines 220 - 225, etc.) that should be instead placed in a table or in the Supplmental Materials. They do not need to be in the body of the manuscript.

Thank you for your suggestion. I have removed some of the modeling details from the manuscript's main text and retained some basic explanations.

14.Section 3 Conditional Validation: where is the description and figure of condition 1?

I have added the relevant image and description in the text. Please see Figure 6.

15.I suggest combining Figs 6 - 9 into into one figure and replacing the text describing each condition (lines 255 - 269) with a table containing a summary of this information.

The modifications have been completed as suggested.

16.Would it be possible to use a different color scheme for Fig 10 to clearly show the KVS effects? It is a little unclear now since the yellow, blue, and green are not easy to differentiate.

I'd prefer to keep the current color scheme and have replaced the figure with a clearer one for you to observe more easily.

17. The authors have not addressed my previous comment: The PLOS data policy requires that authors must make their data fully available without restriction. Please consider hosting the model, real aqueduct topology and parameters, and code on a public repository such as Github or in the Supplemental Materials.

I have added all the data and models in the Supplemental Materials.
