# Supplementary material for: Study on optimization measures for water level fluctuation of large water conveyance aqueducts
Source: PLoS One. 2025 Apr 1;20(4):e0320397. doi: 10.1371/journal.pone.0320397 (PMC11960963; doi:10.1371/journal.pone.0320397)
Supplement: Attachment — Submitted filename: Response_to_Reviewers_auresp_3.docx [file pone.0320397.s004.docx]

Dear Editor and Reviewer,

We sincerely thank the editor and all reviewers for their valuable feedback that we have used to improve the quality of our manuscript. The reviewer comments are laid out below in italicized font and specific concerns have been numbered. Our response is given in normal font and changes/additions to the manuscript are given in the blue text.

**Reviewer 2**

**Comment 1:***In response to my previous comment: "Abstract line 22: "a conical tail pier performs better in eliminating water surface fluctuations": performs better than what other tail pier shapes?" the authors have changed the line in the abstract to "conical tail pier performs better than prismy tail pier in eliminating water surface fluctuations." Are you sure "prismy" is the right word? It does not seem like standard English and you do not mention the word "prismy" anywhere else in the manuscript.*

**Response:**Thank you for your suggestion. I have corrected the mistake and replaced "prismy tail pier" with "platform shaped tail pier."

**Comment 2:***Line 27: why did you choose 15 and 35-meter conical tail pier specifically? Are these common tail pier lengths? Please either generalize the text (e.g., just say short or long) or explain why you chose these numbers.*

**Response:**The intention here is only to express the difference between relatively longer and relatively shorter, and more appropriate modifications have already been made in the text. The following is the revised content.

Furthermore, by comparing the total head loss at the outlet tapering section, it is found that a shorter（e.g.,15-meter） conical tail pier incurs less head loss than a longer（e.g.,35-meter） conical tail pier, indicating that a smaller conical tail pier is more effective. This suggests that shorter tail piers are generally more effective in reducing head loss and improving flow stability.

**Comment 3:** *Thank you for adding more references. I think the Introduction is now more comprehensive and explanatory. However, I still recommend finding and adding credible references to strengthen at least the following sentences because they introduce important definitions and examples:*

*- both the first and second sentences in the Introduction (line 36-38, line 38-40).*

*- Line 51-56*

**Response:**I have added the relevant references to both the first and second sentences in the Introduction and provided a more detailed explanation of the original sentences. The specific content is as follows:

The Karman Vortex Street was first described by von Kármán as a phenomenon where “two parallel rows of vortices alternating in direction are shed in the wake of an obstacle in a fluid with moderate Reynolds numbers.” The wind-induced damage incident of the Tacoma Narrows Bridge in the United States was an accident caused by the Karman Vortex Street. The shedding frequency of the Karman Vortex Street was close to the natural frequency of the bridge deck, leading to resonance. As a result, the amplitude of the vibrations gradually increased, ultimately causing structural instability and collapse.

The original text - Lines 51-56 have also been supplemented with relevant references, namely [4,5,6,7].

**Comment 4:***While the Introduction is more comprehensive now, it is still very disjointed. E.g., in lines 52-56, the authors claim that one can apply a number of practical engineering measures to suppress the generation of the Karman Vortex Street. This sentence is immediately followed by what I believe is a description of the authors' testing scenario. Examples of practical engineering measures only appear starting line 102. In the middle of these two sections is the seemingly random placement of a section on studies on open channel turbulence. I stress, just like in my previous round of comments, that the authors should carefully revisit the format of the Introduction. The content is now stronger than it was previously, but this ordering is confusing and hard to follow.*

**Response:**Thank you very much for your suggestions; this is an important issue. I have adjusted the order of the relevant content, connecting the practical engineering research more closely with the previous section, and moved the research on open channel turbulence after the practical engineering research. I have also added an explanation of the transition section. The content of the transition section is as follows:

Besides the practical application studies in water conveyance engineering, theoretical analysis of open channel turbulence also provides an important foundation for understanding the flow characteristics of aqueducts. The anisotropy of open channel turbulence and the impact of free surfaces on turbulence intensity are directly related to the flow stability in the transition sections of aqueducts. Therefore, it is necessary to combine the research findings on open channel turbulence to further explore the mechanisms of flow fluctuations in aqueducts.

**Comment 5:***Line 151: add a reference for Rhino.*

**Response:**I have added a reference for Rhino, and the specific content is as follows.

Rhino is preferred over other software because it works with NURBS (Non-Uniform Ration B-spline), mathematical representations of 3D geometry that can accurately describe any shape, from the simplest 2D line, circle, arc, or curve to the most complex organic freeform surfaces or solids.

**Comment 6:***Thank you for creating Table 3. I suggest following PLOS One table formatting requirements to make the table a little easier to read and minimize the number of revisions going forward. https://journals.plos.org/plosone/s/tables Adding cell borders will help. You could also make it simpler to read by removing the long sentences in "Condition Description" and instead creating separate columns for length, upstream higher bottom width, upstream lower bottom width, etc. I will leave that up to you.*

**Response:**Thank you for your suggestion. I have modified all the tables in the manuscript according to the formatting requirements of PLOS ONE.

**Comment 7:***Lines 395-398: I'm afraid I don't understand this wording. Do you mean that the Karman Vortex Street only causes flow fluctuations in the aqueduct body section and exit transition section and has no effect on the other sections?*

**Response:**As you mentioned, the original sentence was unclear. I have made adjustments to avoid ambiguity. The revised content is as follows:

The impact of the Karman Vortex Street is more pronounced in the body section of the channel, as well as in the transition section near the outlet, where water surface fluctuations are more significant. In contrast, the fluctuations in the transition section at the inlet and the upstream trapezoidal channel section are almost negligible.

**Comment 8:***Lines 406-409: I suggest rewording this. "The effect is good" is not suitable language for an academic journal.*

**Response:**Thank you very much for your suggestion. I have made the revisions, and the revised content is as follows：

Conical tail piers can eliminate Karman Vortex Street, and when the upstream and downstream lengths are short, the head loss is minimal. The turbulent energy dissipation rate and Froude number are also reduced, resulting in more stable water flow, thus improving the overall hydraulic performance.

**Comment 9:***Line 410-411: please connect these lines back to the Karman Vortex Street.*

**Response:**This section is forward-looking and is not suitable for inclusion in the conclusion. I have removed the corresponding content.

**Comment 10:***Regarding references: Thank you for improving the references. Again, to minimize further revisions, I suggest that you closely follow the PLOS One reference requirements for every single reference: https://journals.plos.org/plosone/s/submission-guidelines#loc-references You will notice that the required ICMJE reference style requires abbreviated journal names, e.g., Measurement Science and Technology should probably be written as Meas Sci Technol (please double check that). The PLOS One guidelines also specify how to write references for articles not written in English.*

**Response:**Thank you very much for your valuable suggestions. I have carefully revised the reference format in accordance with the guidelines provided by PLOS ONE.

**Comment 11:***Since the journal does not copyedit accepted manuscripts, the language should be significantly improved before it can be published. I suggest submitting the paper to an English-speaking third party to improve the grammar and choice of words used throughout the paper.*

**Response:**Thank you very much for your suggestions. I have submitted my paper to a native English speaker specializing in hydraulics, and with their assistance, I have significantly enhanced the overall language quality of the manuscript.
